# Supplementary material for: Psychometric Properties of the Work Ability Index in Health Centre Workers in Spain
Source: Int J Environ Res Public Health. 2021 Dec 9;18(24):12988. doi: 10.3390/ijerph182412988 (PMC8700918; doi:10.3390/ijerph182412988)
Supplement: Supplementary file 1 [file ijerph-18-12988-s001.zip › ijerph-1475648-supplementary.pdf]

Supplementary Table S1. Work ability index.

Indicator	Scale	Scores
1. Subjective estimation of present work ability compared with lifetime best	1-10	0 = very poor<br>10 = very good
2. Subjective work ability in relation to both physical and mental work demands	2-10	2 = very poor<br>10 = very good
3. Number of diagnosed diseases*	1-7	1 = 5 or more diseases<br>2 = 4 diseases<br>3 = 3 diseases<br>4 = 2 diseases<br>5 = 1 disease<br>7 = no disease
4. Subjective estimation of work impairment due to disease	1-6	1 = fully impaired<br>6 = no impairment
5. Sickness absence during the past year	1-5	1 = 100 days or more<br>2 = 25-99 days<br>3 = 10-24 days<br>4 = 1-9 days<br>5 = 0 days
6. Own prognosis of work ability over the next two years	1, 4, 7	1 = hardly able to work<br>4 = not sure<br>7 = fairly sure
7. Psychological resources (enjoyment of daily tasks, alertness and life spirit, optimism for the future)	1-4	1 = very poor<br>4 = very good

*The original WAI3 contains a list of 51 diseases, we used a shortened list of 14 diseases as described by Freyer et al. [1].

Supplementary material S2: Transcultural and content validation

For transcultural validation of the WAI questionnaire, a systematic process was initially used that consisted of two stages: (1) cultural adaptation consisting of direct translation, synthesis, reverse translation and consolidation by an expert panel; (2) questionnaire validation consisting of two stages: 2.1. analysis of applicability, and of face and content validity; and 2.2. psychometric validity: evaluation of internal consistency, and construct and criterion validity.

1. Cultural adaptation phase:

This consisted of questionnaire translation based on its original English version (WAI), whilst striving to maintain its structure through the following procedure:

a- *Direct translation*: Firstly, translation of the instrument into Spanish was performed by two independent bilingual translators. One had knowledge of the aims and concepts of the questionnaire, whilst the other did not.

b- Translation synthesis: Translations were compared, and translators identified and debated any discrepancies until a consensus was reached through collaboration with the research team.

c- *Reverse translation:* The synthesised version of the questionnaire agreed upon by the translators was then translated back into the original language by another two independent bilingual translators. These translators were "blind" to the original version of the questionnaire and the study objectives.

d- Consolidation by expert panel: A multidisciplinary team composed of experts in the fields of methodology and work health, in addition to the translators who participated in

the earlier processes, discussed the various versions of the questionnaire until a preliminary final version was obtained. This version was submitted to validation according to the stages described below.

2. Content validation of the Spanish version of the questionnaire.

This was conducted according to the following stages:

2.1. Analysis of applicability, face validity and content validity:

2.1.1. Analysis of applicability/acceptability and content validity within SAS workers:

The questionnaire was administered to a gender-balanced sample of 30 SAS workers of different professional categories and ages, who attended a routine health check-up in the occupational health department of the centres that participated in the present study. These individuals were selected through convenience sampling. They were requested to provide prior study information and sign an informed consent form, before then being asked to complete the preliminary version of the questionnaire. Further, through structured interviews with scripted questions, participants were asked their opinion about any aspect that was difficult for them to understand. This structured interview was conducted by trained members of the research team. Missing data, and ceiling and floor effects were analysed according to dimensions and items. Questions that posed problems to at least 15% of participants were reviewed, in addition to those with more than 10% missing data, or ceiling and floor effects higher than 15%. This helped us to identify potential improvements to the redaction, design and manageability/format of the questionnaire. From this, a version was produced which was submitted for evaluation by the expert panel via the Delphi technique.

2.1.2. Expert consultation techniques via an online questionnaire:

A total of 10 working health specialists, 6 occupational health experts and 4 experts trained in other specialties participated in this technique. All participants had more than 3 years of experience. The review was carried out via email. Participating individuals expressed their opinions through an evaluation matrix. This included different questions about the questionnaire in general and requested that respondents scored each question according to two criteria: clarity and applicability. The scale on which scores were given ran through 1 (a lot), 2 (a little) and 3 (not at all). Criteria were taken from the validation study of the Cuban version of the instrument [2]. Questions were considered valid when expert panel responses had an agreement level of 70% or higher for category 1 responses (a lot). Further, a column was included where experts could suggest recommendations to improve the formulation of each question.

References

1. Freyer, M.; Formazin, M.; Rose, U. Factorial validity of the work ability index among employees in Germany. *J. Occup. Rehabil.* **2018**, *29*, 433–442, doi:10.1007/s10926-018-9803-9
2. López Pumar, G.M.; del Castillo Martin, N.P.; Viera, A.O. Validation and reliability of the Cuban version of the work ability index (WAI) questionnaire. *Rev. Cuba. Salud Trab.* **2011**, *12*, 29–34.
